# CircUBAP2 Promotes MMP9-Mediated Oncogenic Effect via Sponging miR-194-3p in Hepatocellular Carcinoma

**DOI:** 10.3389/fcell.2021.675043

**Published:** 2021-06-22

**Authors:** Boqiang Liu, Yuanshi Tian, Mingyu Chen, Hao Shen, Jiafeng Xia, Junjie Nan, Tingting Yan, Yifan Wang, Liang Shi, Bo Shen, Hong Yu, Xiujun Cai

**Affiliations:** ^1^Zhejiang Provincial Key Laboratory of Laparoscopic Technology, Sir Run Run Shaw Hospital, School of Medicine, Zhejiang University, Hangzhou, China; ^2^Department of Diagnostic Ultrasound and Echocardiography, Sir Run Run Shaw Hospital, School of Medicine, Zhejiang University, Hangzhou, China; ^3^State Key Laboratory for Diagnosis, National Clinical Research Center for Infectious Diseases, The First Affiliated Hospital, School of Medicine, Zhejiang University, Hangzhou, China; ^4^Department of General Surgery, Sir Run Run Shaw Hospital, School of Medicine, Zhejiang University, Hangzhou, China; ^5^Zhejiang Minimal Invasive Diagnosis and Treatment Technology Research Center of Severe Hepatobiliary Disease, Zhejiang University, Hangzhou, China; ^6^Zhejiang Research and Development Engineering Laboratory of Minimally Invasive Technology and Equipment, Zhejiang University, Hangzhou, China; ^7^Zhejiang University Cancer Center, Zhejiang University, Hangzhou, China

**Keywords:** hepatocellular carcinoma, proliferation, migration, invasion, circUBAP2, miRNA sponge, miR-194-3p, MMP9

## Abstract

**Background:**

The physiological regulatory functions of circRNAs have become a topic of intensive research in recent years. Increasing evidence supports a significant role of circRNAs during cancer initiation and progression, including hepatocellular carcinoma (HCC).

**Materials and Methods:**

A bioinformatics analysis from three independent Gene Expression Omnibus (GEO) databases was performed to profile and screen the dysregulated circRNAs in HCC. RT-qPCR was used to examine the expression level of circUBAP2 in HCC and adjacent non-tumor tissues. Then, proliferation assays (CCK8 and colony formation) and migration assays (transwell and wound healing) were performed to examine effect of circUBAP2 *in vitro*. Immunoprecipitation, RNA pulldown, FISH, and dual-luciferase reporter assay was conducted to explore the circUBAP2-related mechanism for regulating HCC progression. Moreover, a mouse xenograft model and a mouse lung metastasis model confirmed the effect of circUBAP2 *in vivo*.

**Results:**

In this study, we found a novel circRNA: circUBAP2, which was identified by bioinformatics analysis. Among 91 HCC patients, circUBAP2 was significantly upregulated in HCC tissues, and negatively correlated with aggressive clinical characteristics and prognosis. Functional assays demonstrated that circUBAP2 promoted cell proliferation, colony formation, migration, and invasion *in vitro*. Moreover, circUBAP2 enhanced tumor growth and pulmonary metastasis *in vivo*. Mechanistically, circUBAP2 acts as a competing endogenous RNA (ceRNA) for miR-194-3p, a tumor suppressor in HCC. We confirmed that MMP9 was direct target for miR-194-3p, which was regulated by circUBAP2.

**Conclusion:**

CircUBAP2 plays a significant role in promoting HCC via the miR-194-3p/MMP9 pathway and could serve as a promising prognostic biomarker and novel therapeutic target for HCC patients.

## Introduction

Hepatocellular carcinoma (HCC) is the sixth most common malignancy and the third leading cause of cancer-related death globally (Sung et al., 2021). Despite aggressive and multimodal treatment regimens including surgery, chemotherapy, intervention, targeted therapy and immune therapy, the overall survival of HCC patients remains poor (Bray et al., 2018). HCC and other solid tumors have the common characteristic of genetic and phenotypic heterogeneity, which also pose a daunting challenge for diagnosis, treatment and patient care (Llovet et al., 2016). Therefore, it is critical to further dissect the mechanisms of HCC progression in order to develop novel therapeutics to better handle it.

Circular RNAs (circRNAs) are a novel class of non-coding, single-stranded RNAs characterized with covalently closed loop structures with neither a 5′ to 3′ polarity nor a polyadenylated tail, usually produced from back-splicing of exons of pre-mRNAs (Chen, 2020). And the use of 5′ and 3′ splice sites for back-splicing can compete with linear RNA splicing, in which splicesome is considered to play a significant regulatory role (Ashwal-Fluss et al., 2014; Jeck and Sharpless, 2014). A recent study has reported that silence of many core spliceosomal components, such as SF3b and SF3a complexes, resulted in preferred circRNA expression (Liang et al., 2017). This suggested that depleting spliceosomal factors or chemically inhibiting spliceosomal activities could make the transition from canonical splicing to back-splicing (Liang et al., 2017). On the other hand, in addition to core spliceosomal factors, a group of splicing regulators are required for circRNA expression, such as NF90/NF110 (Li et al., 2017). Despite these recent advances, how exactly the spliceosome is involved in back-splicing still remains incompletely understood.

Previously, circRNAs were regarded as by-products of splicing and thought to occur with low abundance. However, increasing evidence supports a significant role of circRNAs during cancer initiation and progression, including in HCC (Hansen et al., 2013; Han et al., 2017; Fang et al., 2018; Yu et al., 2018; Chen et al., 2019; Hu et al., 2019; Li et al., 2019; Chen Q. et al., 2020; Ding et al., 2020; Xu H. et al., 2020; Xu Y. et al., 2020). The critical regulatory functions of circRNAs include miRNA sponges, protein translation templates and the regulation of parental expression. Current research suggests circRNAs usually function as sponges to specific microRNA (miRNA) (Hu et al., 2019; Li et al., 2019), some play as scaffold between proteins (Liu B. et al., 2020; Xu Y. et al., 2020), and a few of them can be translated into peptide to perform biological functions (Legnini et al., 2017; van Heesch et al., 2019).

Matrix metalloproteinase-9 (MMP9) is a significant matrix proteinase which plays vital roles in progression of malignancy (Garg et al., 2011; Yan et al., 2013; Lu et al., 2015; Chen G. et al., 2020). MMP9 can cleave many extracellular matrix (ECM) proteins to regulate ECM remodeling, and then affects invasion, metastasis and angiogenesis of tumors (Egeblad and Werb, 2002).

In the present study, we found that circUBAP2 was significant upregulated in HCC and increased circUBAP2 level was a predictive indicator of poor prognosis in HCC patients. Moreover, we used HCC-bearing xenograft models and several HCC cell lines to explore biological roles and mechanisms of circUBAP2 in HCC progression and metastasis. We found that circUBAP2 upregulates MMP9 by sponging miR-194-3p to promote HCC progression. Therefore, upregulated circUBAP2 is implicated as a potential therapeutic target in HCC.

## Materials and Methods

### Clinical Sample

A cohort of 91 randomly selected HCC clinical samples (since February 2006) and a total of 30-paired HCC and adjacent non-tumor tissues (since September 2018) were collected from patients undergoing initial hepatectomy in Sir Run-Run Shaw Hospital (SRRSH), Zhejiang University (Hangzhou, China). All of the collected tumor tissues were pathologically diagnosed as HCC. The adjacent non-tumor tissues were at least 2 cm away from the edge of lesion and no HCC cells were found in pathological examination. All of these samples were quickly frozen to −80°C in tissue cryopreservation solution. We reviewed pathology records to identify samples with confirmed HCC. This study conformed to the principles of the Declaration of Helsinki and was approved by the Ethics Committee of the SRRSH (Hangzhou, China).

### Cell Lines and Culture

Human normal liver cell line LO2 and human HCC cell lines, including HepG2, HA22T, MHCC97H, HCCLM3 and Huh7 were purchased from the Type Culture Collection of the Chinese Academy of Sciences (Shanghai, China). All the cell lines were cultured in Dulbecco’s Modified Eagle’s Media (Thermo Fisher Scientific, United States) supplemented with 10% fetal bovine serum (FBS, Thermo Fisher Scientific, United States) in a 5% CO_2_ humidified incubator at 37°C. All the experiments were performed within 3 months of resuscitation and the cell passage was less than 15 generations from initial resuscitation.

### Tumor Tumorigenesis and Metastasis Model *in vivo*

To establish HCC-bearing xenograft models, HA22T cells were stably transfected with overexpression-circUBAP2 (oe-circUBAP2) or control vector, and Huh7 cells were stably transfected with short hairpin RNA-circUBAP2 (sh-circUBAP2) or control vector. After that, 6 weeks old male mice were injected with HCC cells into armpits subcutaneously (5 × 10^6^ cells per mouse). Tumor length and width were measured using a caliper every week for 28 days, and tumor volume was calculated as: length × (width)^2^ × 0.5. Finally, the mice were sacrificed in the fourth week after injection. And we also used 6 weeks old male mice to establish the metastasis models by injecting approximately 2 × 10^6^ sh-circUBAP2-HCCLM3 or sh-NC-HCCLM3 cells into the lateral tail vein. Mice injected with HCC cells were sacrificed after 8 weeks and lung samples were isolated for hematoxylin and eosin (H&E) staining to detect the lung metastasis. All animal experiments were performed humanely in compliance with guidelines reviewed by the Animal Ethics Committee of the Biological Resource Centre of the Agency for Science, Technology and Research, Zhejiang University.

### RNA Extraction and Quantitative Real-Time Polymerase Chain Reaction (RT-qPCR)

Total RNAs either from cells or frozen tissues were extracted by using Trizol reagent (Invitrogen, United States), while FFPE RNA Kits (Omega, United States) were applied to isolate total RNAs from paraffin sections of HCC patients. 2 μg of total RNA was subjected to reverse transcription according to the manufacturer instructions of reverse transcription systems (Bio-Rad, United States). qRT-PCR was conducted using a 7500 Real-Time PCR system (Applied Biosystems by Life Technologies, United States) with SYBR green (Yeasen, China) to determine the expression levels of targets. Expression levels of circRNAs and mRNAs were normalized to the expression levels of GAPDH, while small RNA RNU6 (U6) was used for miRNA. The detecting primers for circRNAs were designed based on its head-to-tail junction, and all primer sequences were displayed in Supplementary Material.

### Actinomycin D Transcription Inhibition Assay

Approximately, 2.0 × 10^5^ Huh7 cells were seeded in 12-well plates and cultured until complete adherence. After that, actinomycin D (Abcam, United States) was added to the culture media at a final concentration of 2 μg/ml. Then, the cells in wells were collected for RNA extraction at 0, 3, 6, 12, and 24 h, respectively. The RNA samples were subjected to reverse transcription and RT-qPCR systems.

### RNase R Treatment

Total RNA (2.5 ug) extracted from Huh7 cells was treated with 10U RNase R (Geneseed, China) at 37°C for 30 min. The expression levels of circUBAP2 and mUBAP2 were measured by RT-qPCR.

### Fluorescence *in situ* Hybridization (FISH)

Fluorescent *in Situ* Hybridization Kit (Ribo, China) was used according to the manufacturer’s protocols. To analyze the localization of circUBAP2, HCC cells were hybridized in hybridization buffer with Cy3-labeled probes specific to the circUBAP2 back-splice region (RiboBio, China) at 37°C protected from light overnight. To analyze the co-localization of circUBAP2 and miR-194-3p, Cy3-labeled circUBAP2 probes and FAM-labeled miR-194-3p probes (TsingKe, China) were used. DAPI was used for staining cell nuclei. The images were acquired by using Leica SP8 confocal fluorescence microscope (Leica, Germany).

### Transwell Assay

For migration assay, cells (Approximately 8 × 10^4^ for Huh7 and 2 × 10^4^ for HA22T) in serum-free medium were plated into the upper chambers of 8 mm-pore-size polycarbonate membrane filters (Corning, United States), and culture media with 10% FBS was placed into the lower chambers for a certain period (48 h for Huh7 and 12 h for HA22T) in a humidified incubator at 37°C in 5% CO_2_. As for invasion assay, before seeding the cells, 10 mL of Matrigel (BD, United States) was dissolved in 50 mL serum-free proper culture medium, applied to upper chamber, and put into the incubator for 2 h. The cells remaining in the upper chamber were carefully removed using a cotton swab, and cells that migrated to the lower membrane surface were fixed in 4% paraformaldehyde and stained with crystal violet. Cells were counted in five randomly selected microscopic fields (200 × magnification of the microscope) in each experiment, and the experiment was performed three times.

### Wound-Healing Assay

Culture-insert 2 well (Ibidi, Germany) was put in 12-well plates first, and then HCC cells (approximately 2 × 10^4^ for Huh7 and 1 × 10^4^ for HA22T) were seeded into the well. The cell migration images were photographed with an inverted microscope at 0 and 24 h after scratching.

### CCK-8 Assay

Cell viability was examined using a Cell Counting Kit-8 (Dojindo, Japan) according to the manufacturer’s methods. Approximately 2.5 × 10^3^ cells were plated in 96-well plates. The time points for assessment were set at 0, 24, 48, and 72 h after the cells were plated. The cells were treated with 10 μl of CCK-8 solution and incubated for 1 h, after which OD values were measured at 450 nm using a microplate reader (BioTek, United States).

### RNA Immunoprecipitation (RIP) Assay

To prepare antibody-coated beads, 20 μL Protein A + G Agarose beads were incubated with 1–5 mg antibody or control IgG in 500 μL lysis buffer supplemented with Protease Inhibitor Cocktail, Phosphatase Inhibitor Cocktail Panobinostat and Methylstat at 4°C overnight. Then, the beads were washed twice with lysis buffer and kept on ice. Cell lysates were precleared using 10uL Protein A + G Agarose beads by rotating at 4°C for 3 h. The precleared lysates were transferred to tubes with antibody-coated beads, and then rotated tubes at 4°C for 3–5 h. The protein-captured beads were washed with lysis buffer for three times. RNA extraction from the beads was collected by using Trizol according to the manufacturer’s instructions for later detection. Also, nuclear and cytoplasmic RNA isolation were by using PARIS^TM^ Kit (Thermo Fisher Scientific) according to manufacturer’s instruction.

### Biotin-Labeled Pull-Down Assay

For circRNA pulldown assays, cells lysates were prepared by using IP lysis buffer (Beyotime, China) and followed the manufacturer’s instruction of Pierce^TM^ Magnetic RNA-Protein Pull-Down Kit. Briefly, washed Pierce Streptavidin Magnetic Beads were incubated with cell lysate at 4°C for 1 h for preclearance. The 5′ biotin-labeled circRNA probe incubated with the beads at RT for 10 min for immobilization. Then, the biotinylated beads were incubated with cell lysate at 4°C overnight. The biotinylated beads were magnetically separated and washed five times. For RT-qPCR assays and circRNA purification, Trizol reagent was used to extract the total RNA from the beads.

### Dual-Luciferase Reporter Assay

The wild-type (WT) and mutated-type (MUT) sequences of the binding sites between circUBAP2 and miR-194-3p and the WT and MUT sequences of the binding sites between MMP9 and miR-194-3p were cloned into dual-luciferase reporter vectors (GP-miRGLO) by GenePharma (Shanghai, China). Cells were seeded into 12-well plates and cultured for 24 h and then transfected with dual-luciferase reporter vector and miR-194-3p mimics or negative control (NC) mimics. After 48 h of transfection, the firefly and renilla luciferase activities were measured consecutively by a Dual-Luciferase Reporter Assay System (Promega, United States) on a microplate reader (BioTek, United States). The firefly luciferase activities were normalized to Renilla luciferase activities.

### MMP9 Zymography Assay

Prepare an 8% acrylamide gel containing gelatin. Then load sample to each well, including a protein molecular weight marker in one well. Run the gel at 20 mA until good band separation is achieved. Wash the gel 2 × 30 min with washing buffer. Rinse for 5–10 min in incubation buffer at 37°C with agitation. Replace with fresh incubation buffer and incubate for 24 h at 37°C. Stain the gel with staining solution for 30 min to 1 h. Rinse with ddH2O. Incubate with destaining solution until bands can clearly be seen. Areas of enzyme activity appear as white bands against a dark blue background.

### Statistical Analysis

The applicable statistical methods were used depending on the type of data. The Student’s t-test was used for comparisons between groups. One-way analysis of variance (ANOVA) for multiple comparisons was used to detect differences amongst the various treatments. Kaplan–Meier method were used for survival analysis. Linear correlations were evaluated by Pearson correlation analysis. All data from three separate experiments at least are presented as mean ± SD. Differences were considered significant for *P*-values less than 0.05. ns means *P* < 0.05, ^∗^*P* < 0.05, ^∗∗^*P* < 0.01, and ^∗∗∗^*P* < 0.001.

## Results

### CircUBAP2 Was Significantly Upregulated in HCC Tissues and Correlated With Poor Prognosis

**FIGURE 1:**
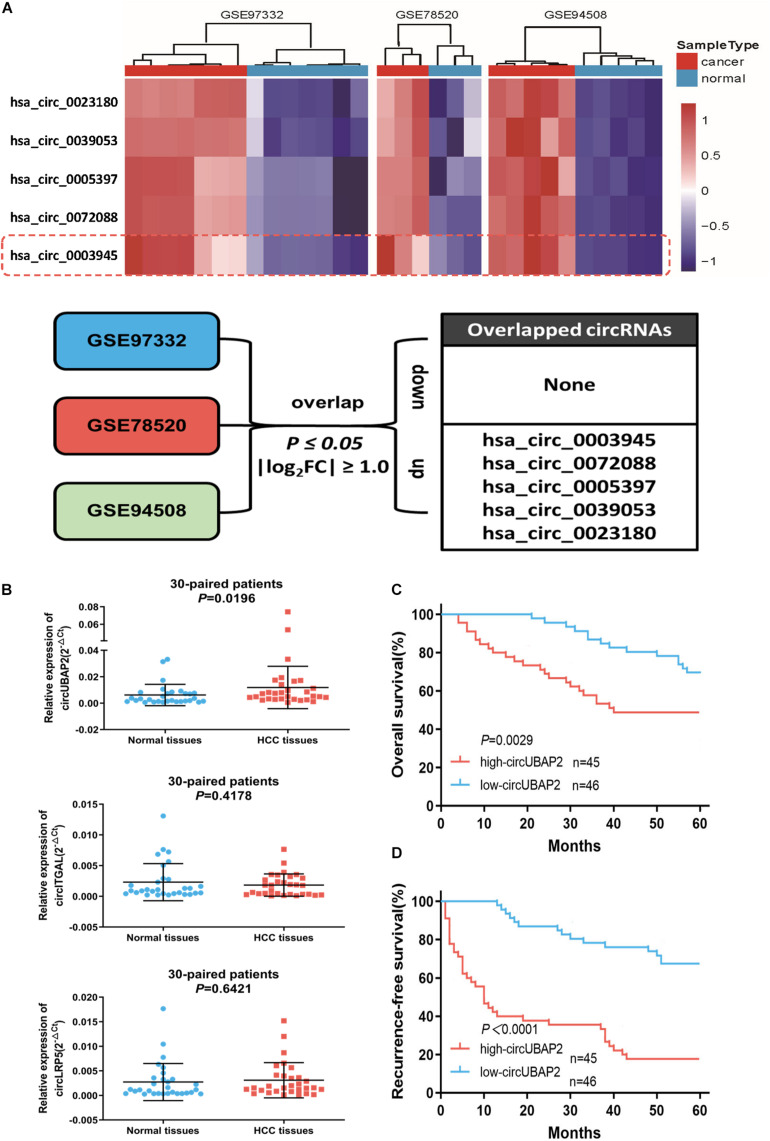
CircUBAP2 was significantly overexpressed in HCC and predicted poor prognosis **(A)**. The overlap of altered circRNAs in GSE97332, GSE78520, and GSE94508 datasets. **(B)** RT-qPCR was used to further validate the differences in the expression of three candidate circRNAs in 30 HCC tissues and paired normal tissues. **(C,D)** Kaplan–Meier curves showing overall survival **(C)** and disease-free survival **(D)** of 91 HCC patients followed up to 60 months. Patients were separated by the median expression level of circUBAP2.

To explore the differentially expressed circRNAs in HCC tissues, we downloaded three microarray gene profiling datasets (GSE97332, GSE78520 and GSE94508) from the Gene Expression Omnibus (GEO) for bioinformatics analysis (Fu et al., 2017; Han et al., 2017; Xiong et al., 2018) (Supplementary Figures 1A,B). The circRNAs with P-value ≤ 0.05 and | log_2_FC| ≥ 1.0 from the t-test were identified as differentially expressed circRNAs. In order to improve the reliability of screening, we only selected the top 250 circRNAs with the most significant differences in expression in each dataset for subsequent combined analysis. Finally, the results showed that 5 circRNAs were consistently upregulated and none were downregulated (Figure 1A). Furthermore, two of up-regulated circRNAs, namely circRHOT1 (hsa_circ_0005397) and circZFR (hsa_circ_0072088), have been proven to play important roles in HCC development, confirming high reliability of the analysis method (Tan et al., 2019; Wang et al., 2019; Yang et al., 2019). Hence, we were interested in evaluating the effect of the other three circRNAs on HCC. Comparisons between 30-paired HCC and adjacent non-tumor tissues showed that transcription of circUBAP2 (hsa_circ_0003945) was significantly increased (*P* = 0.0196), while no significance in the transcription of circITGAL (hsa_circRNA_0039053) or circLRP5 (hsa_circRNA_0023180) were observed (Figure 1B).

We next investigated whether circUBAP2 expression correlates with prognosis in HCC patients. According to the expression level of circUBAP2, 91 HCC cases were divided into two groups: high-circUBAP2 group (*n* = 45, fold-change at least equal to the median) and low-circUBAP2 group (*n* = 46, fold-change up to the median). Results showed that upregulated levels of circUBAP2 were significantly associated with increased tumor size and high tumor recurrence rate (Table 1).

**TABLE 1:** Clinicopathological analyses of the differentially expressed circUBAP2 of 91 primary hepatocellular carcinoma patients.

**Variables**		**CircUBAP2**		***P* value**
		**High (*n* = 45)**	**Low (*n* = 46)**	
Age, years	≤60	28 (62.2)	25 (54.3)	0.5256
	> 60	17 (37.8)	21 (45.7)	
Gender	Male	36 (80.0)	32 (69.6)	0.3357
	Female	9 (20.0)	14 (30.4)	
HBV	No	11 (24.4)	13 (28.3)	0.8127
	Yes	34 (75.6)	33 (71.7)	
Cirrhosis	No	23 (51.1)	27 (68.7)	0.5303
	Yes	22 (48.9)	19 (41.3)	
TNM stage	I, II	37 (82.2)	40 (87.0)	0.5737
	III, IV	8 (17.8)	6 (13.0)	
Tumor size, cm	≤ 5	19 (42.2)	31 (67.4)	0.0208
	> 5	26 (57.8)	15 (32.6)	
Recurrence	No	13 (28.9)	31 (67.4)	0.0003
	Yes	32 (71.1)	15 (32.6)	

*The expression level of circUBAP2 were examined by RT-qPCR. Chi-squared test was used. *P* values < 0.05 were considered statistically significant.*

Moreover, Kaplan-Meier survival curve analysis showed that high-circUBAP2 group had significantly poorer overall survival (OS, *P* = 0.0029) and recurrence-free survival (RFS, *P* < 0.0001) (Figures 1C,D). These data suggested that circUBAP2 plays an important role in HCC progression, and might act as a novel prognostic factor for HCC patients.

### Characterization of CircUBAP2 in HCC

**FIGURE 2:**
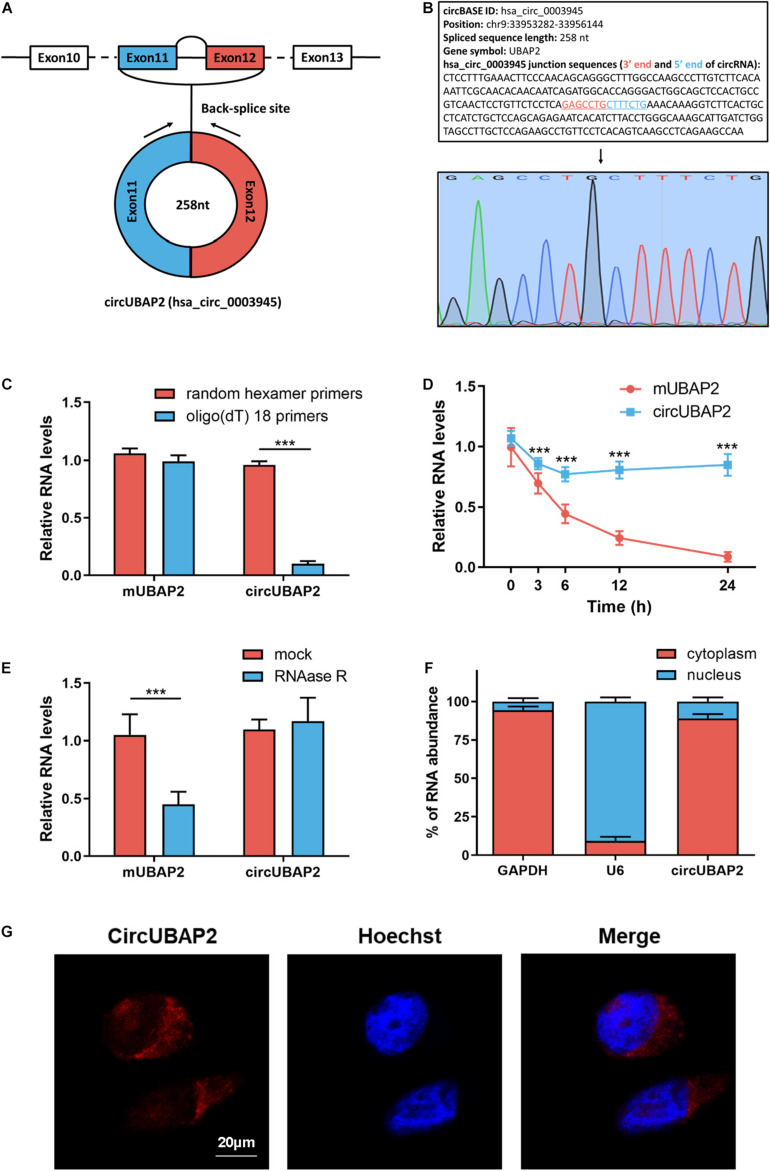
CircRNA characterization of circUBAP2 in HCC. **(A)** The exonic information of circUBAP2 (circBase ID: hsa_circ_0003945) is illustrated as indicated. **(B)** The specific primers of circUBAP2 were validated by Sanger sequencing. The black arrow indicates the backsplice site. **(C)** Random hexamer or oligo (dT) 18 primers were utilized for reverse transcription assays. The relative RNA levels were examined by RT-qPCR and normalized to those generated using random hexamer primers. **(D)** The relative RNA levels were examined by RT-qPCR after treatment with actinomycin D at the indicated time points. **(E)** The relative RNA levels were examined by RT-qPCR after treatment with RNase R or mock in total RNAs. **(F)** The cellular distribution of circUBAP2 was analyzed by cellular RNA fractionation assays. GAPDH and U6 were used as cytoplasmic and nuclear positive controls, respectively. **(G)** The cellular distribution of circUBAP2 was analyzed by fluorescence *in situ* hybridization (FISH). Scale bar = 20 μm. ***P < 0.001.

Bioinformatics web of circRNAs (circBASE^1^) showed that circUBAP2 is an exonic circRNA that is cyclized with the 11 and 12 exons of the UBAP2 gene and the spliced length of circUBAP2 is 258 nt (Figure 2A). Subsequently, we designed circUBAP2-specific primer to confirm the existence of circUBAP2, the RT-qPCR product was analyzed by agarose gel electrophoresis and Sanger sequencing to validate the head-to-tail splicing (Figure 2B).

Next, we applied oligo (dT)18 or random hexamer primers in reverse transcription experiments of total RNAs from Huh7 cells. The result showed that oligo (dT)18 primers could not efficiently guide circUBAP2 reverse transcription, while mUBAP2 reverse transcription was successfully guided by both oligo (dT)18 and random hexamer primers (Figure 2C). Then, we measured the half-life of circUBAP2 and mUBAP2 in Huh7 cells by using actinmycin D (an inhibitor of transcription). The results illustrated that circUBAP2 was more stable than mUBAP2 (Figure 2D). Furthermore, circUBAP2 showed higher resistance to digestion by RNase R (a highly processive 3′–5′ exoribonuclease that digests linear RNAs) than mUBAP2 (Figure 2E). Collectively, these results mentioned above demonstrated that circUBAP2 is stable and harbors a loop structure without ployA-tail. Additionally, RT-qPCR after cellular RNA fractionation and fluorescence *in situ* hybridization (FISH) assays showed the predominant cytoplasmic distribution of circUBAP2 in Huh7 cells (Figures 2F,G).

### CircUBAP2 Promotes the Migration, Invasion, and Proliferation of HCC Cells *in vitro*

**FIGURE 3:**
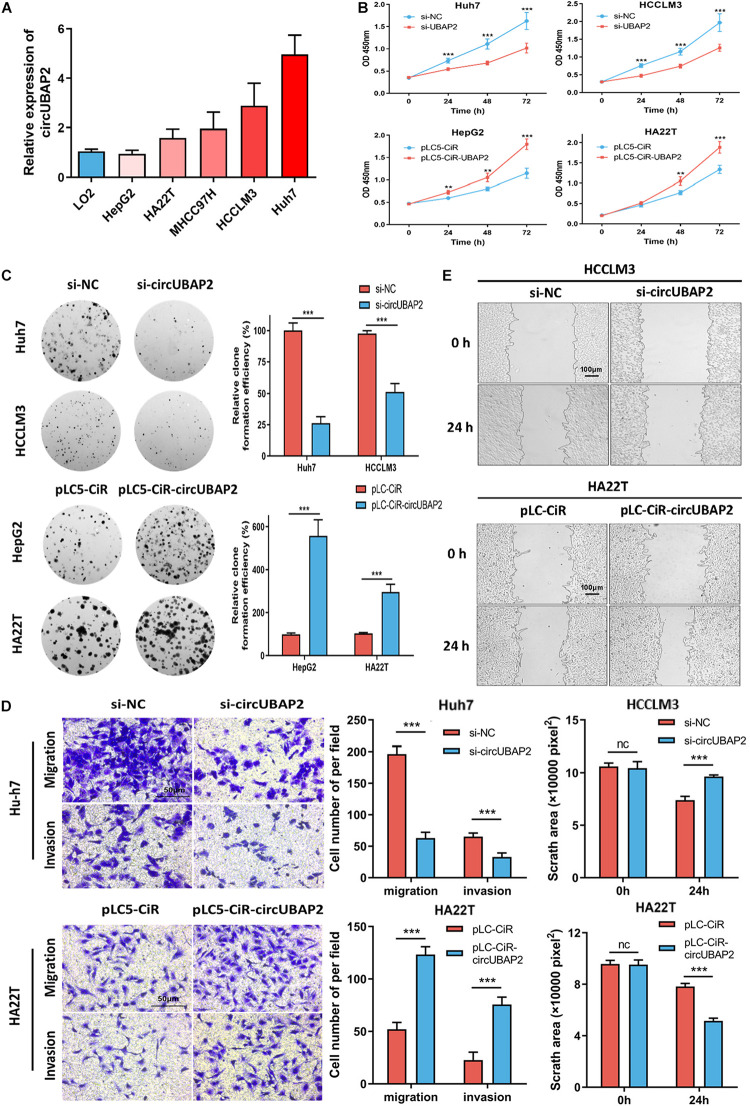
CircUBAP2 promotes HCC proliferation, migration and invasion *in vitro*. **(A)** The expression levels of circUBAP2 in normal liver cell line and several HCC cell lines were analyzed by RT-qPCR. **(B,C)** CCK-8 and colony formation assays showed that circUBAP2 improved the proliferation of HCC cells. **(D,E)** Transwell (scale bar = 50 μm) and wound-healing assays (scale bar = 100 μm) showed that circUBAP2 promoted the migration and invasion of HCC cells. The black lines in **(E)** represent frontlines of the migrating cells. ns means *P* > 0.05, **P > 0.01 and ***P > 0.001.

First, we evaluated the circUBAP2 expression level in several HCC cell lines. The results showed that circUBAP2 expression levels was higher in four HCC cell lines (Huh7, HCCLM3, MHCC97H, and HA22T) and lower in HepG2, compared to LO2 (a normal liver cell line) (Figure 3A). We selected Huh7/HCCLM3 for circUBAP2 knocking-down and HepG2/HA22T for overexpression according to endogenous level. The efficacies of circUBAP2 modulations were verified respectively and also, mRNA of UBAP2 remained intact (Supplementary Figures 2A,B).

Then we applied CCK-8 and colony formation assays to evaluate the effects of circUBAP2 on cell growth. The results demonstrated that knocking-down of circUBAP2 suppressed cell proliferation and colony formation in Huh7 and HCCLM3 cells (Figures 3B,C). Moreover, migration and invasion were also significantly inhibited after circUBAP2 silence (Figure 3D). Similarly, this result was also verified by wound-healing assay (Figure 3E). Consistently, we received coherent results after overexpressing circUBAP2 in HepG2 and HA22T cells (Figures 3B–E). Together, these results indicated that circUBAP2 could promote the proliferation, migration and invasion of HCC cells *in vitro*.

### CircUBAP2 Functions as a Sponge of miR-194-3p

**FIGURE 4:**
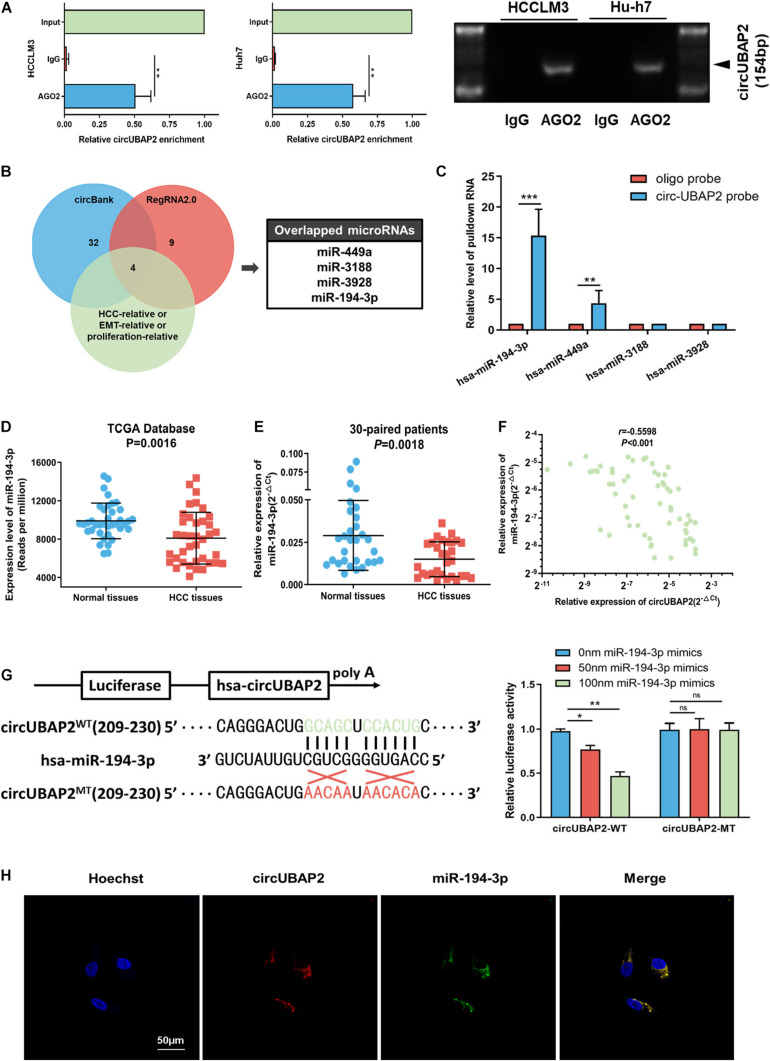
CircUBAP2 functions as a sponge for miR-194-3p. **(A)** RNA immunoprecipitation (RIP) and RT-qPCR assays were conducted to analyze the binding of circUBAP2 to the AGO2 protein. **(B)** Bioinformatics analysis was performed for predicting and filtering possible miRNA candidates. **(C)** CircRNA-pulldown assays were performed using circUBAP2-specific biotin probe and oligo probe. RT-qPCR assays were performed to analyze potential miRNAs associated with circUBAP2. The enrichment of potential miRNAs was normalized to that of the control probe. **(D)** TCGA database shows that the expression of miR-194-3p is reduced in HCC tissues. **(E)** The expression level of miR-194-3p was further assessed in 30 HCC tissues and paired normal tissues. **(F)** CircUBAP2 level was negatively correlated with miR-194-3p levels, which was validated by RT-qPCR in 30 HCC tissues. The correlation was evaluated by Pearson correlation analysis. **(G)** Wild-type (WT) and mutated-type (MT) sequences of the putative binding sites between circUBAP2 and miR-194-3p. Luciferase activity in HA22T cells co-transfected with luciferase reporters containing circUBAP2 sequences with wild-type or mutated miR-194-3p binding sites and mimics of miR-194-3p or controls. **(H)** The FISH experiments showed that circUBAP2 and miR-194-3p colocalized in the cytoplasm. Scale bar = 50 μm. ns means *P* > 0.05, *P > 0.05, **P > 0.01 and ***P > 0.001.

Considering that circRNAs have been reported to function as sponges for miRNAs and that circUBAP2 is stable and located in the cytoplasm, we investigated the sponging ability of circUBAP2. First, we conducted RNA immunoprecipitation (RIP) of AGO2 in Huh7 and HCCLM3 cells and analyzed the relative expression levels of circUBAP2 enriched by anti-AGO2 or IgG immunoprecipitation pellet. Compared with IgG group, anti-AGO2 group showed obviously stronger ability of circUBAP2 enrichment, which suggested that circUBAP2 may functions as a sponge of miRNAs (Figure 4A).

Hence, we applied the bioinformatics program (circBank^2^ and RegRNA2.0^3^) for predicting and filtering possible miRNA candidates. Additionally, to improve the reliability of bioinformatics analysis, these miRNA candidates should also have been previously reported metastasis-relative, proliferation-relative, or HCC-relative at least. Based on these criteria, four miRNAs (miR-449a, miR-3188, miR-3928, and miR-194-3p) were chosen, which shared binding sites with circUBAP2 (Figure 4B). To determine the functional miRNA targets of circUBAP2, we designed a 5′-terminal-biotinylated-circUBAP2 probe that could pull down circUBAP2 specifically in HCC cells. The levels of these four candidate miRNAs showed that only miR-194-3p and miR-449a were significantly enriched by the circUBAP2-specific probe (Figure 4C). Furthermore, we investigated the expression levels of these two candidate miRNAs in HCC and normal tissues from miRNA microarray data (The Cancer Genome Atlas, TCGA^4^). The results showed that, compared with normal tissues, the miR-194-3p was significantly decreased in HCC (*P* = 0.0016), which was consistent with our results tested from 30-paried cases (*P* = 0.0018) (Figures 4D,E). And Pearson correlation analysis showed a significant inverse correlation between circUBAP2 and miR-194-3p in 60 HCC cases (*r* = −0.5598, *P* < 0.001) (Figure 4F). Additionally, the TCGA data indicated that higher level of miR-194-3p correlated with better prognosis in HCC (Supplementary Figure 3A). However, the expression levels of miR-449a are higher in HCC tissues than those in normal tissues (*P* < 0.001) (Supplementary Figure 3B). Taken together, miR-194-3p is a potential target of circUBAP2 in HCC.

To further confirm this hypothesis, a dual luciferase reporter assay was performed in HCC cells. We observed that miR-194-3p mimics attenuated the luciferase activity significantly in circUBAP2-wildtype (circUBAP2-WT) group, and demonstrated a dose-dependent effect. Whereas miR-194-3p mimics had no influence on luciferase activity in circUBAP2-mutant (circUBAP2-MT) group (Figure 4G). Moreover, the co-localization of circUBAP2 and miR-194-3p was found by FISH in HCC cells (Figure 4H).

### CircUBAP2 Modulates the Progression of HCC via the miR-194-3p-MMP9 Pathway

**FIGURE 5:**
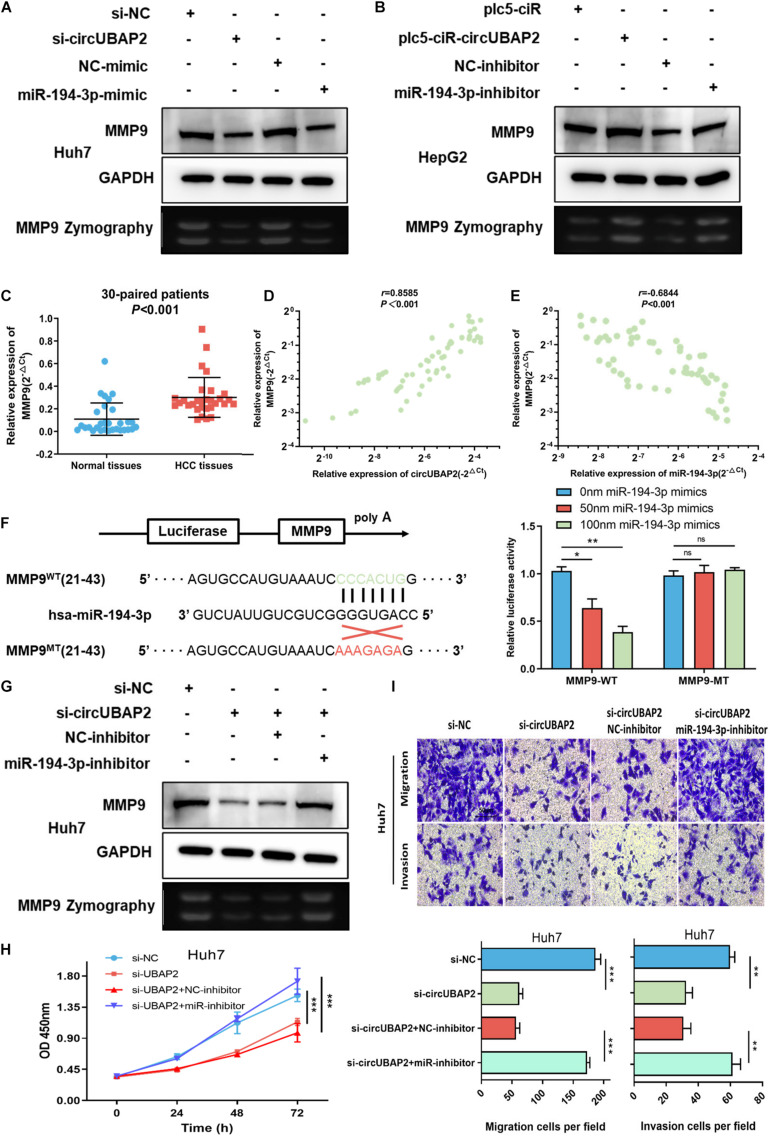
CircUBAP2 promotes the progression of HCC through the miR-194-3p-MMP9 pathway. **(A,B)** Western-blot (WB) and zymography experiments examined the expression levels of MMP9 in HCC cells after silencing or overexpressing circUBAP2 or miR-194-3p. **(C)** The expression level of MMP9 was further assessed in 30 HCC tissues and paired normal tissues. **(D)** MMP9 level was positively correlated with circUBAP2 levels, which was validated by RT-qPCR in 30 HCC tissues. The correlation was evaluated by Pearson correlation analysis. **(E)** MMP9 level was negatively correlated with miR-194-3p levels, which was validated by RT-qPCR in 30 HCC tissues. The correlation was evaluated by Pearson correlation analysis. **(F)** WT and MT sequences of the putative binding sites between miR-194-3p and MMP9 mRNA. Luciferase activity in HA22T cells co-transfected with luciferase reporters containing MMP9 sequences with wild-type or mutated miR-194-3p binding sites and mimics of miR-194-3p or controls. **(G)** WB and zymography experiments analyzed the expression of MMP9 in HCC cells after treatment with si-circUBAP2 alone or combined with si-circUBAP2 and miR-194-3p inhibitors. **(H)** CCK-8 assays analyzed the proliferation of HCC cells after treatment with si-circUBAP2 alone or combined with si-circUBAP2 and miR-194-3p inhibitors. **(I)** Transwell assays analyzed the migration of HCC cells after treatment with si-circUBAP2 alone or combined with si-circUBAP2 and miR-194-3p inhibitors. ns means *P* > 0.05, *P > 0.05, **P > 0.01 and ***P > 0.001.

There is accumulating evidence that miR-194-3p directly targets numerous oncogenes, including PRC1, RUNX2, DPAK1, MECP2, and MMP9, and suppresses tumor progression in different malignancies (Abuduaini et al., 2018; Zhou et al., 2018, 2020; Xu et al., 2019; Yi et al., 2019). Therefore, circUBAP2 might play a pro-tumor role by modulating expression of miR-194-3p targeting oncogenes mentioned above. To test this hypothesis, we overexpressed and silenced the expression of circUBAP2 in HCC cells and found that only MMP9 was markedly negatively regulated by circUBAP2 as expected (Supplementary Figure 4A). Moreover, both reduction of circUBAP2 and upregulation of miR-194-3p could decrease the protein expression levels of MMP9 (Figure 5A). In contrast, the protein expression of MMP9 was significantly upregulated after overexpressing circUBAP2 or silencing miR-194-3p (Figure 5B). The MMP9 zymography experiment obtained consistent results as above (Figures 5A,B).

According to previous reports, the MMP9 gene acts as an important oncogene and promotes cancer progression in HCC (Egeblad and Werb, 2002; Yan et al., 2013; Lu et al., 2015). The TCGA data indicated that HCC tissues exhibited increased expression of MMP9 (Supplementary Figure 4B). Again, this result was verified in 30-paired cases (*P* < 0.001) (Figure 5C). In addition, higher level of MMP9 correlated with earlier distant metastasis and higher tumor stage, which predicted poorer prognosis in HCC (Supplementary Figures 4C–E). Furthermore, analyzed with Pearson correlation analysis, a significant positive correlation between circUBAP2 and MMP9 was detected (*r* = 0.8585, *P* < 0.001), while the correlation between miR-194-3p and MMP9 was negative (*r* = −0.6844, *P* < 0.001) in 60 HCC cases (Figures 5D,E). To further examine the interaction between miR-194-3p and MMP9, we conducted a dual luciferase reporter assay. The results showed co-transfection of miR-194-3p mimics and MMP9-wildtype (MMP9-WT) vectors markedly attenuated the luciferase activity. In contrast, co-transfection of miR-194-3p mimics and MMP9-mutant (MMP9-MT) vectors demonstrated no effect on luciferase activity (Figure 5F).

In order to strengthen our claims, a series of rescue assays were performed. We demonstrated that miR-194-3p inhibitors could significantly reverse the downregulation of MMP9 induced by silencing circUBAP2 (Figure 5G). Functionally, transwell and CCK-8 assays revealed that cell proliferation suppressed by silencing circUBAP2 was blocked by miR-194-3p inhibitor treatment (Figures 5H,I). Finally, we overexpressed MMP9 in circUBAP2 knocking down cells, which successfully rescued carcinogenic features (Supplementary Figures 5A,B).

### CircUBAP2 Promotes HCC Progression *in vivo*

**FIGURE 6:**
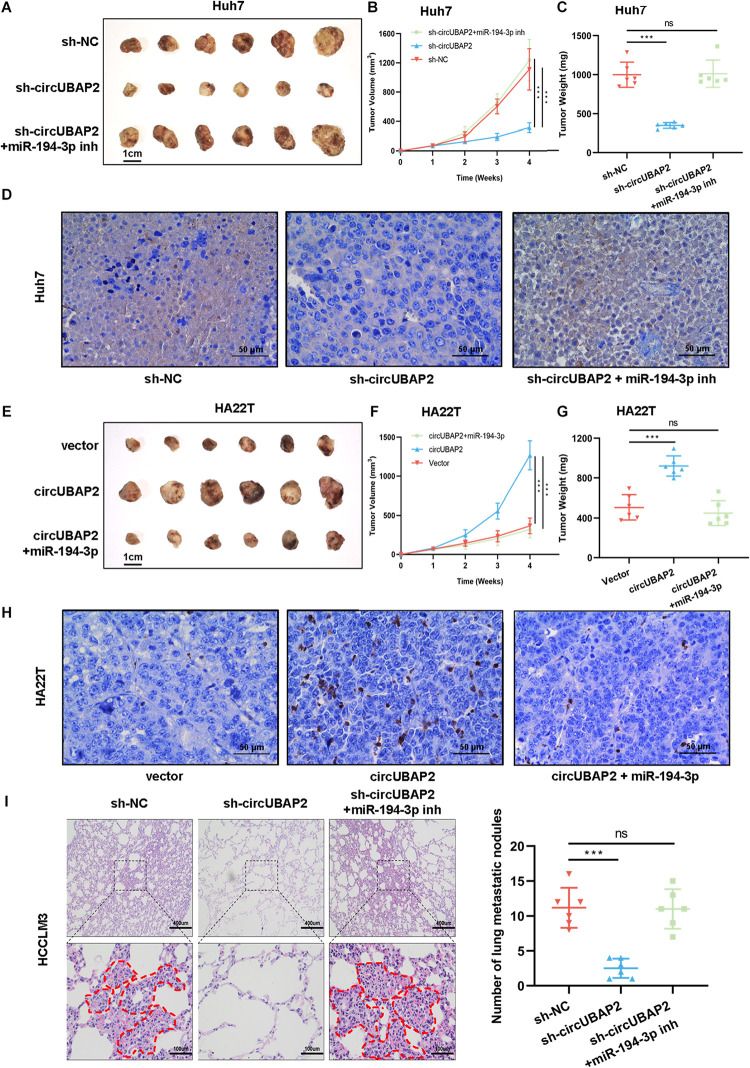
CircUBAP2 promotes the progression of HCC *in vivo*. **(A)** Images of subcutaneous xenografts of sh-NC, sh-circUBAP2 and sh-circUBAP2 with miR-194-3p inhibitors Huh7 cells in nude mice (*n* = 6). **(B)** The volume of subcutaneous xenografts induced by Huh7 cells in nude mice was recorded every week for 4 weeks (*n* = 6). **(C)** The Huh7 tumor weights were calculated after removing the tumors (*n* = 6). **(D)** Immunohistochemical detection of MMP9 expression in Huh7-derived tumor tissues **(E)** Images of subcutaneous xenografts of oe-vector, oe-circUBAP2 and oe-circUBAP2 with miR-194-3p mimics HA22T cells in nude mice (*n* = 6). **(F)** The volume of subcutaneous xenografts induced by HA22T cells in nude mice was recorded every week for 4 weeks (*n* = 6). **(G)** The HA22T tumor weights were calculated after removing the tumors (*n* = 6). **(H)** Immunohistochemical detection of MMP9 expression in HA22T-derived tumor tissues **(I).** HE staining and number of the HCCLM3-derived lung metastatic nodules at the endpoint (*n* = 6). ns means *P* > 0.05 and ***P > 0.001.

To further evaluate the effect of circUBAP2 on HCC tumorigenesis and progression *in vivo*, HCC cells transfected with sh-circUBAP2/overexpression vectors and miR-194-3p mimics/inhibitors were injected subcutaneously into nude mice (6 mice per group). The tumor volume was measured once a week for 4 weeks after injection. Compared with the NC group, the tumor volume and weight were significantly lower in the sh-circUBAP2 group, and this could be reversed by co-transfection of the miR-194-3p inhibitors (*P* < 0.001, Figures 6A–C). WB experiment and immunohistochemistry for MMP9 confirmed that silence of circUBAP2 downregulated MMP9 *in vivo* and miR-194-3p inhibitors could reverse this phenotype (Figure 6D and Supplementary Figure 6A). As expected, overexpression circUBAP2 could promote the growth and metastasis of HCC cells *in vivo*, whereas co-transfection of the miR-194-3p mimics reversed these effects (*P* < 0.001, Figures 6E–G). Immunohistochemistry detection and WB experiment also showed consistent results (Figure 6H and Supplementary Figure 6B).

Moreover, we established mouse lung metastasis model by injecting sh-circUBAP2 and sh-NC HCCLM3 cells into the lateral tail vein of nude mice (6 mice per group). After 8 weeks, the results showed that the number of metastatic nodes was significantly decreased in the sh-circUBAP2 group in comparison with those in the control group, and co-transfection of the miR-194-3p inhibitors could reverse this phenotype (Figure 6I).

**FIGURE 7:**
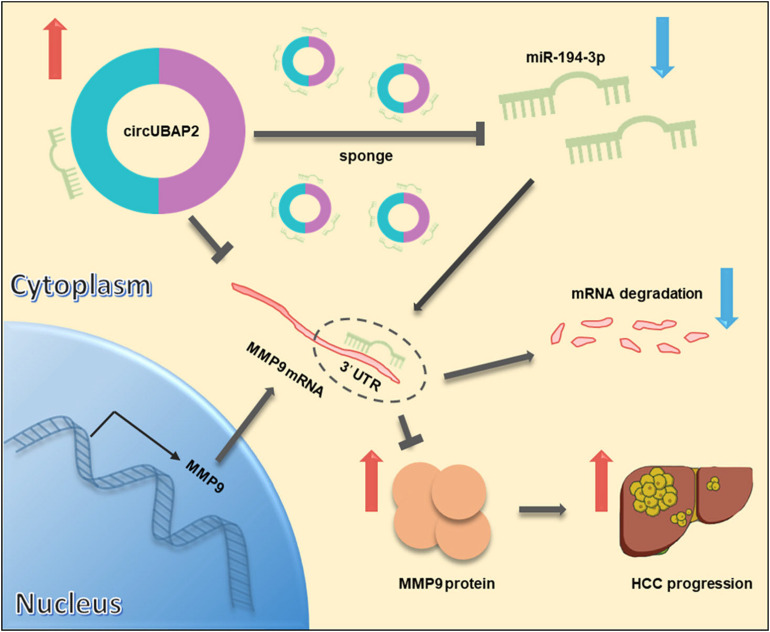
Schematic diagram of the mechanism and function of circUBAP2 in HCC progression. CircUBAP2 promotes the development and progression of HCC through the circUBAP2-miR-194-3p-MMP9 axis.

Taken together, these results demonstrated that circUBAP2 promotes metastasis and proliferation of HCC via sponging miR-194-3p and regulating MMP9 (Figure 7).

## Discussion

The physiological regulatory functions of circRNAs have become a topic of intensive research in recent years. Numerous reports have linked them with HCC, such as circSMARCA5, circMAT2B, and circASAP1, which have distinct roles in HCC progression and were all suggested to function as ceRNAs that antagonize certain microRNAs (Han et al., 2017; Yu et al., 2018; Hu et al., 2019). In this study, we identified a novel circRNA derived from UBAP2, termed circUBAP2, by bioinformatics analysis. We first discovered that circUBAP2 is frequently upregulated in HCC, and its expression significantly correlated with poor patients’ prognosis, indicating its applicability as a novel prognostic biomarker in HCC. High level of circUBAP2 was associated with increased tumor size and high tumor recurrence rate in HCC patients. Functionally, overexpression of circUBAP2 promoted HCC cell proliferation and metastasis *in vitro* and *in vivo*, while knockdown of circUBAP2 reversed these effects, suggesting that circUBAP2 act as an oncogenic factor in HCC.

Many circRNAs contain potential miRNA complementary sites, which suggests that circRNAs can function as miRNA sponge to exert their biological roles through specific circRNA-miRNA-mRNA axis (Chen, 2020). For instance, circSMARCA5 could promote the expression of TIMP3 by sponging miR-181b-5p and miR-17-3p (Yu et al., 2018). CircMAT2B promotes glycolysis and malignancy of HCC by sponging miR-338-3p (Li et al., 2019). Moreover, circASAP1 was observed to regulate cell migration and invasion by sponging miR-326 and miR-532-5p (Hu et al., 2019). In the present study, to explore miRNAs targeted by circUBAP2, we applied circBANK and RgeRNA2.0 to predicate and filter possible miRNA candidates, which should also have been previously reported metastasis-relative, proliferation-relative, or HCC-relative. After filtering out 2 candidates, we further validated their expression levels in HCC tissues by using TCGA database and 30-paired patients’ samples. Ultimately, only miR-194-3p meet the aforementioned criteria, which was also confirmed by RNA pull-down, dual-luciferase reporter and colocalization assays. Previous reports have indicated that miR-194-3p functions as a tumor suppressor and prognostic marker in multiple types of cancers, and directly targets mRNA of multiple oncogenes, including PRC1, RUNX2, DPAK1, MECP2 and MMP9 (Abuduaini et al., 2018; Yi et al., 2019; Zhou et al., 2020). In this study, we confirmed that MMP9, a critical regulator of ECM and tumor microenvironment, is the direct target of the circUBAP2/miR-194-3p axis.

MMP9 belongs to the zinc-dependent endopeptidase family, which can degrade ECM and plays a vital role in tumorigenesis and metastasis (Xia et al., 2016; Jiang et al., 2017; Liu F. et al., 2020; Pan et al., 2020). Xia et al. (2019) reported that MMP9 was a directly target of miR-149 in oral squamous cell carcinoma. Liu F. et al. (2020) observed that MMP9 was overexpressed in bladder cancer and was negatively regulated by miR-491-5p. Our study demonstrated that MMP9 was negatively associated with miR-194-3p, and circUBAP2 acts as a sponge of miR-194-3p, upregulating MMP9 expression in HCC cells (Figure 7). To the best of our knowledge, this is the first study to report the directly regulatory effect of circRNA on MMP9 in HCC.

ECM and its modifications, as an important component of tumor microenvironment, influence many facets of tumor biology including tumorigenesis and tumor metastasis (Mohan et al., 2020). While ECM degradation is considered a key step promoting tumor progression, MMPs family, especially MMP9 plays a significant role in this process (Vandooren et al., 2013). MMP9 functions predominantly as a collagenase to promote tumor invasion and metastasis by degrading type IV collagen, a major component of basement membrane and the ECM (Hamano et al., 2003). It has been reported that MMP9 can also stimulate tumor growth, while cancer cell proliferation is decreased in tumors from MMP9-deficient mice compared with wild-type mice (Coussens et al., 2000). There are several different pathways in which MMPs promote tumor proliferation. Predominantly, MMPs can release the cell-membrane-bound precursors of some growth factors, such as TGF-α (Peschon et al., 1998). Meanwhile, some growth factors sequestered by ECM become available once degraded by MMPs (Manes et al., 1999). Third, the MMPs can also regulate proliferative signals through integrins pathway indirectly (Agrez et al., 1994).

Although our study has conducted an in-depth exploration of the role of circUBAP2 in HCC, there still exist some limitations needing for more detailed investigation. First, due to the restriction of bioinformatics analyses, there may also exist other miRNAs regulated by circUBAP2, which deserves further investigation. Meanwhile, a subset of circRNAs have been reported functioning through other novel mechanisms, such as serving as protein scaffolds, protein translation, and regulating paternal transcripts (Conn et al., 2017; Legnini et al., 2017; Wang et al., 2019). Hence, whether circUBAP2 functions through mechanisms mentioned above in HCC cells remains unclear. Third, though our study revealed the great potential of circUBAP2 to be a promising prognostic biomarker and novel therapeutic target for HCC patients, the conclusion still needs to be verified by clinical data from other medical centers.

## Conclusion

Our results demonstrated that circUBAP2 is significantly upregulated in HCC tissues and negatively correlates with HCC patients’ prognosis. We preliminarily confirmed that circUBAP2 promotes the development and progression of HCC through the circUBAP2-miR-194-3p-MMP9 axis. Our findings indicate that circUBAP2 could serve as a promising prognostic biomarker and novel therapeutic target for HCC patients.

## Data Availability Statement

The datasets presented in this study can be found in online repositories. The names of the repository/repositories and accession number(s) can be found below: https://www.ncbi.nlm.nih.gov/geo/, GSE78520; https://www.ncbi.nlm.nih.gov/geo/, GSE94508; https://www.ncbi.nlm.nih.gov/geo/, GSE97332.

## Ethics Statement

The studies involving human participants and animal were reviewed and approved by the Ethics Committee of the SRRSH (Hangzhou, China). The patients/participants provided their written informed consent to participate in this study.

## Author Contributions

XC, HY, and BS conceived and designed the research. BL, YT, and MC performed most of the experiments. YW and LS provided administrative support and revision suggestion. BL wrote the manuscript with the assistance from HS, JX, JN, and TY. BS and LS collected and provided clinical samples and data. All authors read and approved this version of manuscript.

## Conflict of Interest

The authors declare that the research was conducted in the absence of any commercial or financial relationships that could be construed as a potential conflict of interest.

## Footnotes

1 www.circbase.org

2 www.circbank.cn

3 regrna.mbc.nctu.edu.tw

4 www.cancergenome.nih.gov
