# Supplementary material for: CircUBAP2 Promotes MMP9-Mediated Oncogenic Effect via Sponging miR-194-3p in Hepatocellular Carcinoma
Source: Front Cell Dev Biol. 2021 Jun 22;9:675043. doi: 10.3389/fcell.2021.675043 (PMC8258265; doi:10.3389/fcell.2021.675043)
Supplement: Supplementary file 1 [file Data_Sheet_1.docx]

Supplementary Material

1. **Supplementary Figure Legends**

**Figure S1. Bioinformatics analysis results of altered circRNAs profiling in GSE97332, GSE78520 and GSE94508 datasets.**

**A.** Heatmap of altered circRNAs in GSE97332, GSE78520 and GSE94508 datasets.

**B.** Volcano plot of altered circRNAs in GSE97332, GSE78520 and GSE94508 datasets.

**Figure S2. CircUBAP2 and UBAP2 mRNA levels after overexpression or knockdown of circUBAP2.**

**A.** RT-qPCR results showed that circUBAP2 decreased after knockdown of circUBAP2 in Huh7 and HCCLM3 cells, but there were no changes in UBAP2 mRNA levels.

**B.** RT-qPCR results showed that circUBAP2 increased after overexpression of circUBAP2 in HepG2 and HA22t cells, but there were no changes in UBAP2 mRNA levels.

**Figure S3. The expression level of miR-449a in HCC tissues and normal tissues.**

**A.** Kaplan–Meier curves showed that the expression of miR-194-3p is positively correlated with the prognosis of HCC patients.

**B.** The TCGA database showed that the expression level of miR-449a is higher in HCC tissues than in normal tissues.

**Figure S4. MMP9 is negatively regulated by miR-194-3p and predicts poor prognosis in HCC patients.**

**A.** Knockdown or overexpression miR-194-3p could negatively regulate the expression level of MMP mRNA, but there were no changes in PRC1, RUNX2, DAPK1 and MECP2 mRNA levels.

**B.** The TCGA database showed that the expression level of MMP9 is higher in HCC tissues than in normal tissues.

**C.** Compared with non-metastatic HCC, metastatic HCC has higher expression of MMP9.

**D.** MMP9 expression is positively correlated with HCC tumor grade.

**E.** Kaplan–Meier analysis revealed the prognostic values of MMP9 in 365 HCC tumors.

**Figure S5. The functional rescue assays of MMP9 overexpression**

**A.** Transwell assays showed that MMP9 overexpression significantly rescued the migration of Huh7 cells with circUBAP2 knockdown.

**B.** CCK8 assays showed that MMP9 overexpression significantly rescued the migration of Huh7 cells with circUBAP2 knockdown. Scale bar=50 µm.

**Figure S6. The MMP9 expression was regulated by circUBAP2/miR-194-3p axis *in vivo***

**A and B.** WB experiments showed that MMP9 was negatively regulated by circUBAP2 *in vivo*, and miR-194-3p could reverse the effect of circUBAP2.

**2. Supplementary Materials**

**2.1 Sequence based reagents**

| **Name** | **Sequence** | **Supplier** |
| --- | --- | --- |
| oligo biotinylated probe | aaaTGTGCCGGATGGAGTTCTGCTTTGCCCACTTC | TsingKe |
| CircUBAP2 biotinylated probe | aaaTGTTTCAGAAAGCAGGCTC | TsingKe |
| CircUBAP2 probe with Cy3 conjugated | aaaTGTTTCAGAAAGCAGGCTC | Ribobio |
| miR-194-3p probe with  FAM | aaaCAGATAACAGCAGCCCCACTGG | TsingKe |
| si-circUBAP2 | GAGCCTGCTTTCTGAAACA | Ribobio |
| CircUBAP2 detecting primer F | TCCTCAGAGCCTGCTTTCTG | TsingKe |
| CircUBAP2 detecting primer R | GAGTTGGCTTCTGAGGCTTG | TsingKe |
| CircITGAL detecting primer F | CATTTCATTTCCCGCTCCCG | TsingKe |
| CircITGAL detecting primer R | TGTTACTGGTTGAATAGGAGCACT | TsingKe |
| CircLRP5 detecting primer F | GACGGCTCGTTCCGCCT | TsingKe |
| CircLRP5 detecting primer R | CTCACGTCTGTCCAGTACACG | TsingKe |
| miR-194-3p detecting primer | purchased from TIANGEN BIOTECH CO., LTD | TIANGEN |
| miR-449a detecting primer | purchased from TIANGEN BIOTECH CO., LTD | TIANGEN |
| U6  detecting primer | purchased from TIANGEN BIOTECH CO., LTD | TIANGEN |
| MMP9 detecting primer F | TGTACCGCTATGGTTACACTCG | TsingKe |
| MMP9  detecting primer R | GGCAGGGACAGTTGCTTCT | TsingKe |
| PRC1 detecting primer F | ATCACCTTCGGGAAATATGGGA | TsingKe |
| PRC1  detecting primer R | TCTTTCTGACAGACGGATATGCT | TsingKe |
| DAPK1  detecting primer F | ACGTGGATGATTACTACGACACC | TsingKe |
| DAPK1  detecting primer R | TGCTTTTCTCACGGCATTTCT | TsingKe |
| MECP2 detecting primer F | GCCGAGAGCTATGGACAGCA | TsingKe |
| MECP2  detecting primer R | CCAACCTCAGACAGGTTTCCAG | TsingKe |
| RUNX2  detecting primer F | TGGTTACTGTCATGGCGGGTA | TsingKe |
| RUNX2  detecting primer R | TCTCAGATCGTTGAACCTTGCTA | TsingKe |

**2.2 Antibodies**

| **Name** | **Supplier** | **Cat no.** |
| --- | --- | --- |
| Anti-MMP-9 antibody [EPR22140-154] | Abcam | Cat#ab228402 |
| Anti-GAPDH antibody [6C5] | Abcam | Cat#ab8245 |
| Anti-Argonaute-2 antibody [ERP10411] | Abcam | Cat#ab186733 |
| Rabbit IgG, monoclonal [EPR25A] | Abcam | Cat#ab172730 |

**2.3 Biological samples**

| **Description** | **Source** |
| --- | --- |
| Human HCC tumor tissue and paired liver tissue | Sir Run Run Shaw Hospital |

**2.4 Other (e.g. drugs, proteins, vectors etc.)**

| **Description** | **Source** | **Identifier** |
| --- | --- | --- |
| Hoechst 34580 | Thermo Scientific | Cat#H21486 |
| Rnase R | Epicentral | Cat#RNR07250 |
| Fluorescent in Situ Hybridization Kit | Ribobio | Cat#C10910 |
| CheckMate™/Flexi® Vector Mammalian Two-Hybrid System | Promega | Cat#C9360 |
| FFPE RNA Kit R6954 | Omega Bio-Tek | Cat#R6954-02 |
| Plasmid: PLC-CiR | Geneseed | Cat#GS0108 |
| Plasmid: PLC-CiR-circUBAP2 | This paper | N/A |
| Plasmid: lentiCRISPRv2 | Addgene | Cat#52961 |
| Plasmid: lentiCRISPRv2-circUBAP2 | This paper | N/A |
