# Supplementary figures and images for: CircUBAP2 Promotes MMP9-Mediated Oncogenic Effect via Sponging miR-194-3p in Hepatocellular Carcinoma
Source: Front Cell Dev Biol. 2021 Jun 22;9:675043. doi: 10.3389/fcell.2021.675043 (PMC8258265; doi:10.3389/fcell.2021.675043)

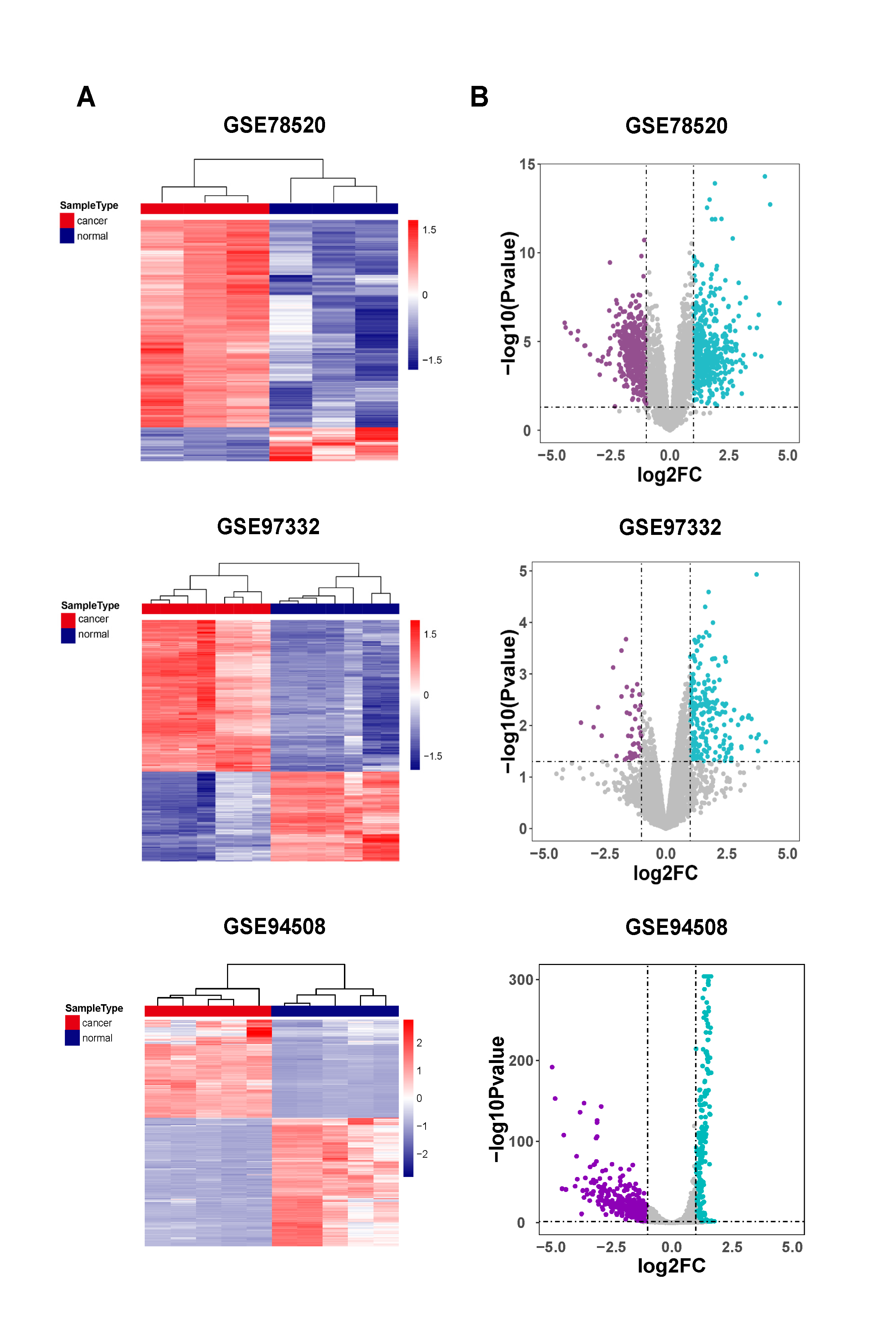

Supplement: Supplementary file 2 [file Image_1.TIF]

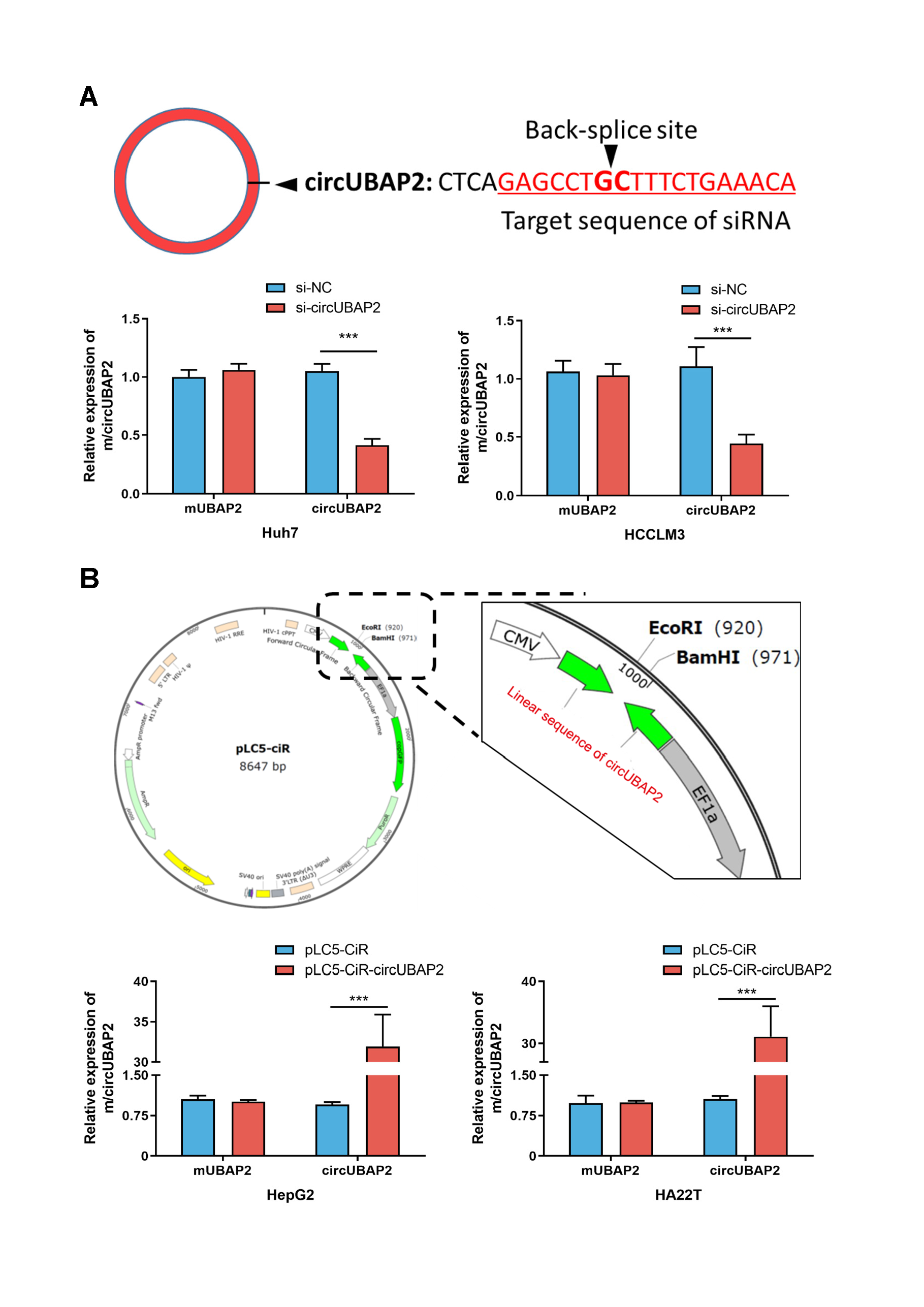

Supplement: Supplementary file 3 [file Image_2.TIF]

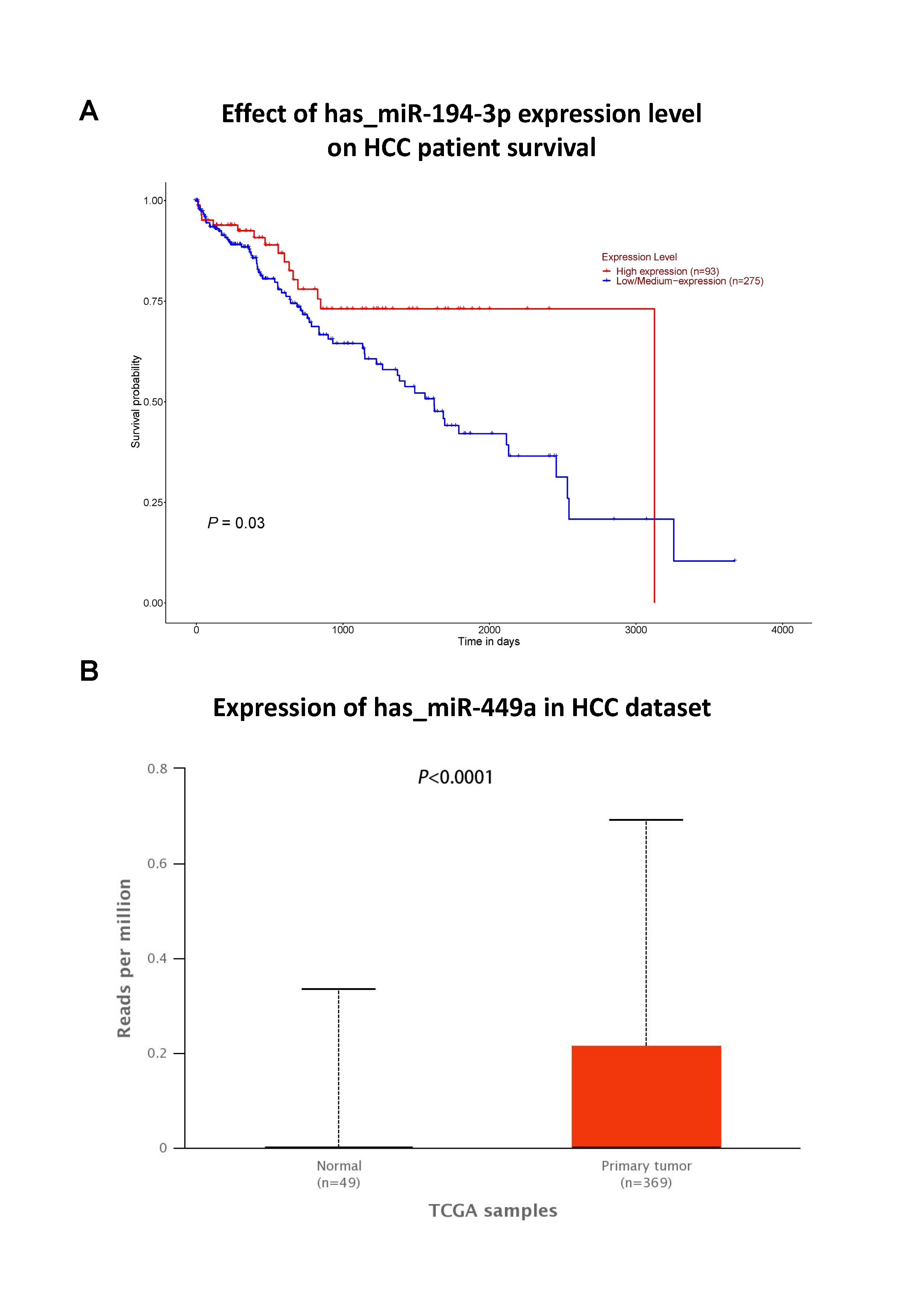

Supplement: Supplementary file 4 [file Image_3.TIF]

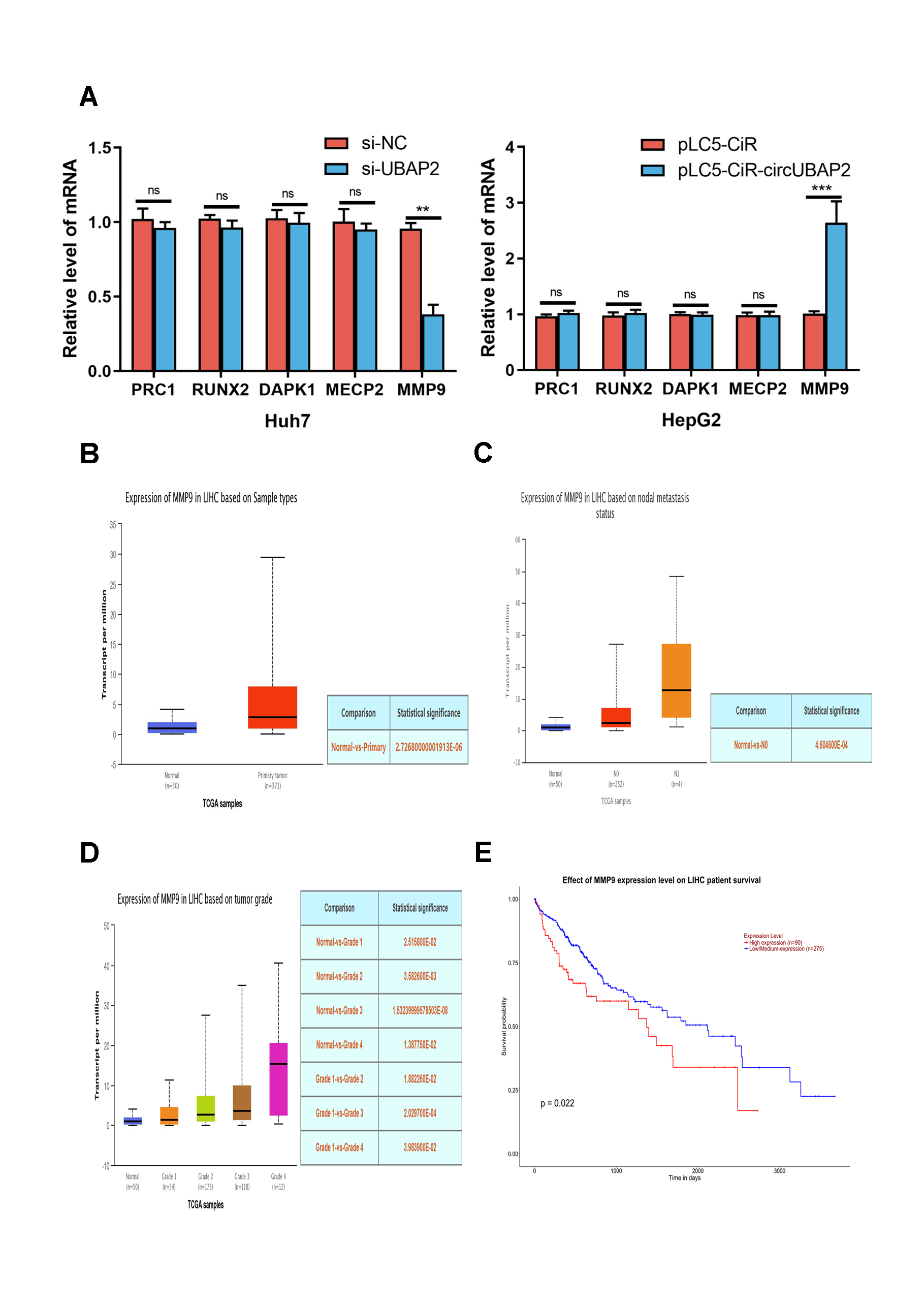

Supplement: Supplementary file 5 [file Image_4.TIF]

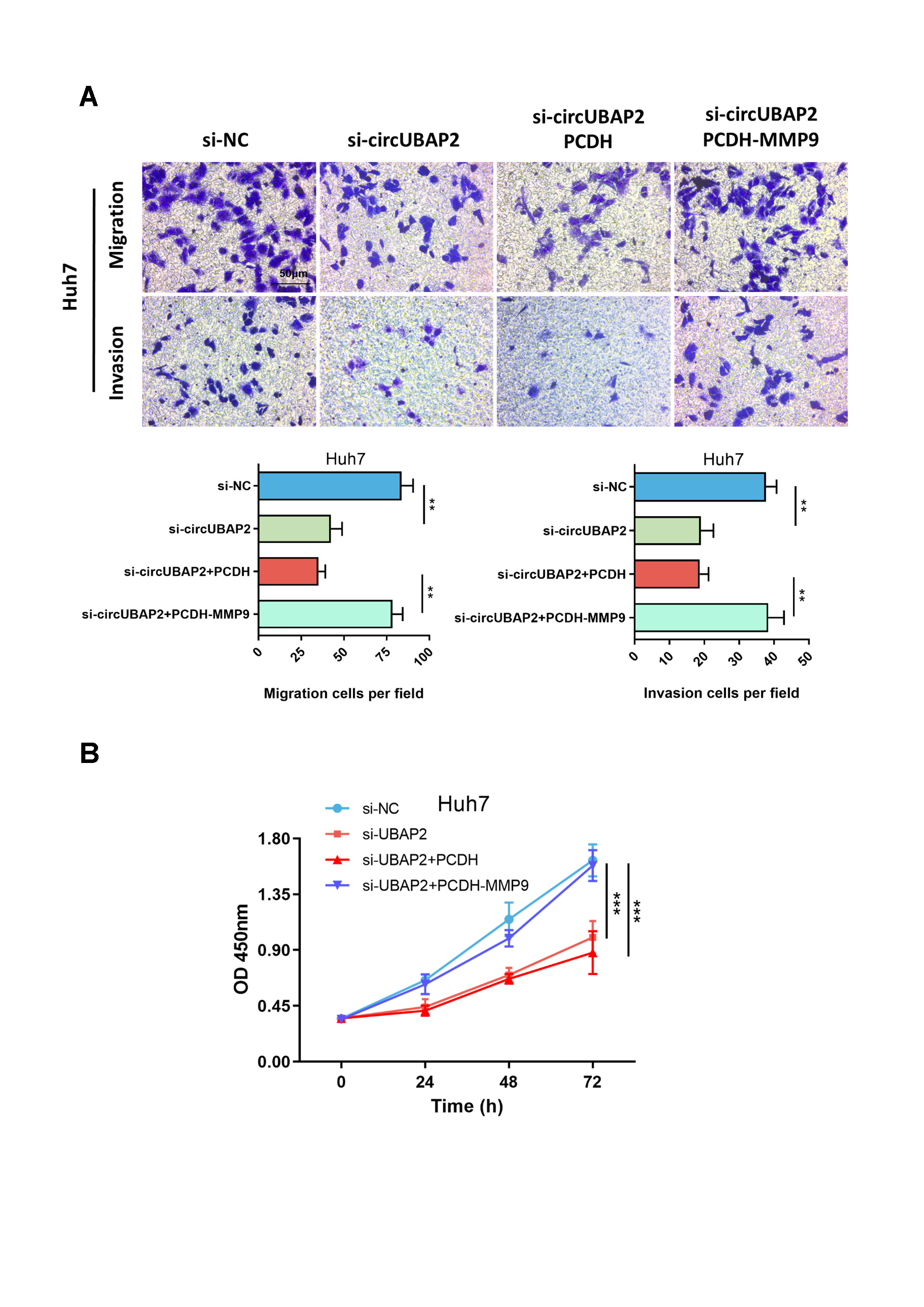

Supplement: Supplementary file 6 [file Image_5.TIF]

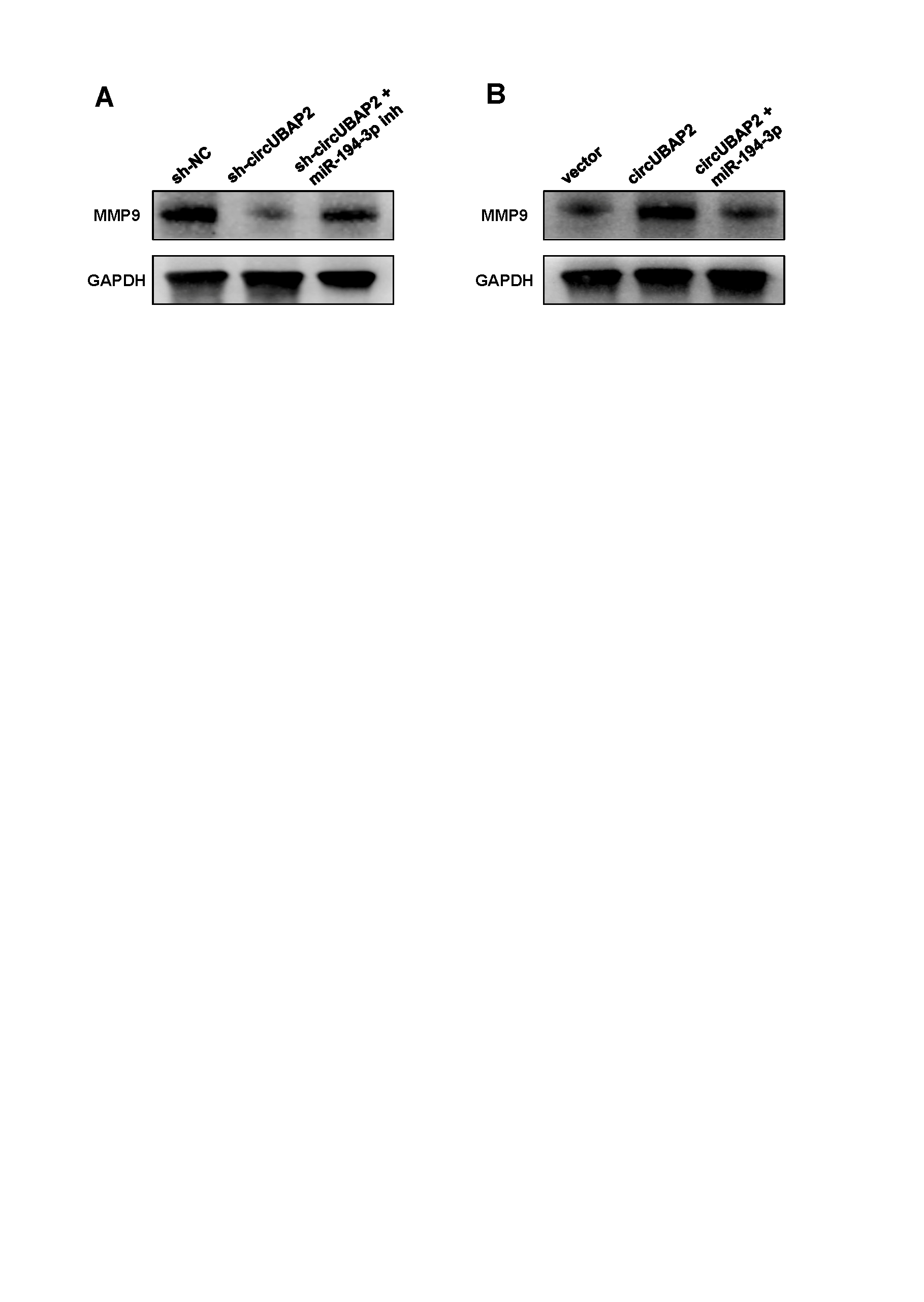

Supplement: Supplementary file 7 [file Image_6.TIF]
